# Features of Cross-Seeding of Wild-Type Alpha-Synuclein and Its Mutant Form A53T Potentially Useful for the Development of Test Systems

**DOI:** 10.3390/life16040675

**Published:** 2026-04-15

**Authors:** Kseniya Barinova, Sofiya Kudryavtseva, Lidia Kurochkina, Sergei Golyshev, Nataliya Kolotyeva, Sergei Illarioshkin, Michail Piradov, Vladimir Muronetz

**Affiliations:** 1 Belozersky Research Institute of Physico-Chemical Biology, Lomonosov Moscow State University, Moscow 119991, Russia; barinovakv@belozersky.msu.ru (K.B.); sofiia.kudriavtceva@gmail.com (S.K.); lpk56@mail.ru (L.K.); sergei.a.golyshev@gmail.com (S.G.); 2 Faculty of Bioengineering and Bioinformatics, Lomonosov Moscow State University, Moscow 119991, Russia; 3 Russian Center of Neurology and Neurosciences, Moscow 125367, Russia; kolotyeva.n.a@neurology.ru (N.K.); illarioshkin@neurology.ru (S.I.); dir@neurology.ru (M.P.)

**Keywords:** alpha-synuclein, seeds, RT-QuIC, amyloid transformation, cross-fibrillation, neurodegenerative diseases

## Abstract

Since the features of cross-seeding of alpha-synuclein forms may affect the sensitivity and specificity of the test systems, we developed a modified approach to obtain alpha-synuclein amyloid seeds with particle sizes from 20 to 50 nm prepared from either the wild-type protein (α-synWT) or its more fibrillation-prone form A53T (α-synA53T). These seeds had optimal properties for subsequent initiation of fibrillation. Our data showed that the elevated efficiency of alpha-synuclein A53T monomer transformation was hardly affected by the type of used seeds, whereas the addition of the seeds obtained from the alpha-synuclein mutant form to wild-type protein monomers had a significantly smaller effect than α-synWT seeds. Transmission electron microscopy data revealed that in the presence of α-synWT seeds the wild-type alpha-synuclein formed long and wide fibrils, while the addition of α-synA53T seeds led to the formation of long, but thin fibrils. Since the lag period of α-synA53T monomer fibrillation was significantly reduced compared to the wild-type protein, the replacing of α-synWT with α-synA53T in current assay systems designed to detect aberrant forms of α-synuclein in biological fluid samples (e.g., RT-QuIC) could substantially cut the time of analysis. In the future, a set of alpha-synuclein mutant forms could be used for the differential diagnosis of synucleinopathies caused by the different mutations of this protein.

## 1. Introduction

The pathological transformation of alpha-synuclein, which leads to the formation of amyloid aggregates, is a key factor in various synucleinopathies, with Parkinson’s disease being the most common and socially significant among them [1,2,3]. The amyloid transformation of alpha-synuclein results in the production of different forms of the protein, including oligomers, protofibrils, fibrils, and Lewy bodies, which are the most evident features of these disorders [4,5,6,7]. The importance of each of these alpha-synuclein intermediate forms in disease pathology is continuously being evaluated. Even so, the critical role of amyloid structure formation by alpha-synuclein in the initiation and progression of Parkinson’s disease is well-established [8,9,10,11]. Therefore, investigating the characteristics of alpha-synuclein’s amyloid transformation is essential for understanding the molecular mechanisms that underlie Parkinson’s disease and other synucleinopathies. This knowledge, in turn, is crucial for both discovering new treatments and developing diagnostic tools. Additional difficulties arise due to the classification of synucleinopathies. There are sporadic forms, in which wild-type alpha-synuclein (α-synWT) is involved in pathological processes, and forms, usually of early manifestation, associated with the production of various alpha-synuclein mutant forms [12,13,14,15].

Recently, new diagnostic methods for synucleinopathies have been developed based on identifying aberrant forms of alpha-synuclein in the cerebrospinal fluid (CSF) of patients. These forms are able to initiate the amyloid transformation of monomeric alpha-synuclein *in vitro* [16,17,18,19,20,21,22,23,24]. The Seed Amplification Assay (SAA), which was initially created to detect prion diseases, has sparked considerable interest in the scientific community. One of the contemporary variants of the SAA is the real-time quaking-induced conversion (RT-QuIC) technology. The principle of these experiments involves the incubation of CSF (containing oligomers, protofibrils, and fibrils of alpha-synuclein) in conditions that promote partial disruption of aggregates. For example, that is done through vigorous shaking or mechanical disruption with added beads. This process results in the multiplication of free sites for their further interaction with monomers of recombinant alpha-synuclein within the amyloid aggregates. When external monomers are added, the aggregates can elongate, then potentially disintegrate during the incubation and form new centers for aggregation. As a result, there is a notable increase in the number of amyloid forms that can be detected using standard techniques, particularly increased fluorescence of thioflavin T (ThT). Such manipulations allow us to detect even tiny quantities of aberrant amyloid forms of alpha-synuclein in patient biological fluids, which typically cannot be revealed by conventional methods like the enzyme-linked immunosorbent assay (ELISA).

Despite the widespread popularity of this method and the presence of numerous re-liable publications, reproducing such protocols faces significant challenges. We assumed that the contradictory information found in the literature data may be due to dropping the unique characteristics of alpha-synuclein amyloid transformation, particularly the cross-seeding interaction between the wild-type and mutant forms. Thus, the use of only wild-type alpha-synuclein monomers in test systems may make it difficult to detect amyloid mutant forms of this protein in biological fluid samples. Moreover, we suggest that the experimental processes could be greatly expedited by using mutant forms of alpha-synuclein as monomers, because these variants tend to have a higher tendency for rapid amyloid formation.

In this study, we explored fibril formation by monomers of the mutant alpha-synuclein A53T (α-synA53T) as well as the wild-type protein. For this purpose, at first, we developed a protocol for obtaining amyloid seeds, which are in fact small amyloid fragments that originate from the amyloid transformation of two distinct forms of alpha-synuclein. Thus, the aim of the work was to investigate the differences in fibril formation between the mutant and wild-type proteins initiated by the addition of different amyloid seeds.

## 2. Materials and Methods

### 2.1. Expression and Purification of Recombinant Wild-Type Alpha-Synuclein and Its Mutant Form A53T

Recombinant human wild-type alpha-synuclein was expressed using *E. coli* BL21 (DE3) bacterial cells transformed with pET33b(+) plasmid containing the SNCA gene with Y136Y (TAC→TAT) substitution. A synonymous substitution was introduced into the gene sequence in order to prevent a previously described translational error for alpha-synuclein [25]. A mutant form of human alpha-synuclein (α-syn A53T) was obtained by site-directed mutagenesis of the SNCA gene, which replaced the 53d alanine residue with a threonine one.

Protein expression was induced by adding IPTG to a final concentration of 1 mM, and cells were incubated for 5 h at 37 °C with shaking.

Briefly, alpha-synuclein was purified using acid precipitation of contaminating proteins by lowering the pH value of the supernatant obtained after bacterial cells’ ultrasonication to 3.0–3.5. Further the pH value of the protein preparation was returned to the neutral range. Then alpha-synuclein was precipitated by adding dry ammonium sulfate to 40% saturation, and the preparation was left for at least 2 h, after which it was dialyzed overnight against bidistilled water at 4 °C. The next day, the protein was lyophilized. Lyophilized alpha-synuclein was stored at −20 °C. Before use, the protein was dissolved in the required buffer, centrifuged for 10 min at 15,000 *g*, and the concentration was determined spectrophotometrically using the extinction coefficient A^0.1%^ (280 nm) = 0.412.

All samples taken during the purification were analyzed by Laemmli SDS-PAGE using 16% separating gel [26].

### 2.2. Alpha-Synuclein Seed Preparation

To obtain seeds, 100 μL of a 1 mg/mL (70 μM) alpha-synuclein solution in PBS buffer, pH 7.4 was mixed with SDS to its final concentration of 0.015%. This sample was put under strong stirring for 10 s, after which the seeds were grown at 42 °C according to the following protocol: incubating for 1 min with stirring at 400 rpm, then left for 1 min rest without stirring—at least 35 such cycles were performed. The seeds’ growth was monitored by continuously measuring thioflavin T fluorescence (excitation at 440 nm, emission at 482 nm; see Section 2.3) and the minimum total time for growth was 70 min.

### 2.3. Alpha-Synuclein Fibrillation

For wild-type alpha-synuclein and its mutant form A53T fibrillation, samples of the following composition were made: protein at a concentration of 0.7 mg/mL (50 μM) in PBS buffer, pH 7.4; a 10-fold excess of thioflavin T; and 0.02% sodium azide. In the case of seeding, the seeds (see Section 2.2) were added to the sample at a 1% of the total protein concentration.

Fibrillation kinetics were analyzed by detecting thioflavin T fluorescence levels in a Greiner 96-well plate with a transparent bottom (Greiner, Kremsmuenster, Austria, non-binding, μClear^®^, black) in 100 μL/well for 72 h. The samples were incubated with constant orbital shaking at 37 °C and fluorescence was measured at 30 min intervals using a CLARIOstar plate reader (BMG LABTECH GmbH, Ortenberg, Germany). Each sample was performed in three independent replicates, and the obtained data were averaged.

Alpha-synuclein fibrillation was also performed in volume. In this case, a 500 µL sample of 0.7 mg/mL (50 µM) protein solution in PBS buffer, pH 7.4, was incubated in glass tubes in the presence of 0.02% sodium azide at 37 °C with constant shaking for 72 h. For the seeding, like the CLARIOstar experiments, the seeds (see Section 2.2) were added to the sample at a 1% of the total protein concentration. At certain time intervals, aliquots were taken to record the kinetics of amyloid particle formation by analyzing the fluorescence spectra of Congo red (see Section 2.4), as well as to determine the kinetics of protein oligomerization and to study the heterogeneity of samples using the DLS method (see Section 2.5).

### 2.4. Congo Red Fluorescence Spectroscopy

Staining with Congo red dye was used to detect the formation of amyloid structures during alpha-synuclein fibrillation (see Section 2.3). The aliquots were taken from samples at certain time intervals and were preincubated with a 10-fold excess of freshly prepared Congo red solution for 20 min at 20 °C. Next, the fluorescence spectra of Congo red were recorded using a FluoroMax^®^-3 spectrofluorometer (Horiba Scientific, Kyoto, Japan) in the range of 520–700 nm (excitation at 497 nm).

### 2.5. Dynamic Light Scattering

Dynamic light scattering (DLS) measurements were performed to study alpha-synuclein oligomerization and the heterogeneity of samples. The experiments were carried out on a Zetasizer Nano-ZS device (Malvern Instruments, Malvern, UK) equipped with a laser (wavelength 532 nm) and 173° optics for detection of scattered intensity to monitor the average particle sizes in the range of 1 to 10,000 nm. The obtained data were analyzed using the parameter “size distribution by volume”. Measurements were made at 25 °C in a 0.1 mL cuvette. Each distribution in the graph was shown as the average value of 5 measurements taken within 75 s.

### 2.6. Transmission Electron Microscopy (TEM)

Samples after alpha-synuclein fibrillation (see Section 2.3) were applied to glow-discharged carbon-coated 300-mesh copper grids. The specimens were then contrasted with 2% aqueous uranyl acetate solution. TEM images were obtained using a JEOL JEM-2100 transmission electron microscope (JEOL, Tokyo, Japan) at an accelerating voltage of 80 kV. TEM studies were carried out at the shared research facility “Electron microscopy in life sciences” at Moscow State University (unique equipment: “Three-dimensional electron microscopy and spectroscopy”).

## 3. Results

We developed a methodology to obtain alpha-synuclein amyloid seeds prepared from either the wild-type protein (α-synWT) or its more fibrillation-prone form A53T (α-synA53T). To effectively stimulate amyloid aggregation of alpha-synuclein monomers, the seeds should be a mixture of relatively small in size amyloid forms (oligomers rather than mature fibrils). Thus, to prevent the formation of large fibrils during seed preparation, we added a small amount of detergent (0.015% SDS) to the sample and applied intensive stirring in a pulsed mode (1 min of shaking followed by 1 min of a rest, see Section 2.2). The optimal temperature for forming seeds with the right features was found to be 42 °C.

**Figure 1:**
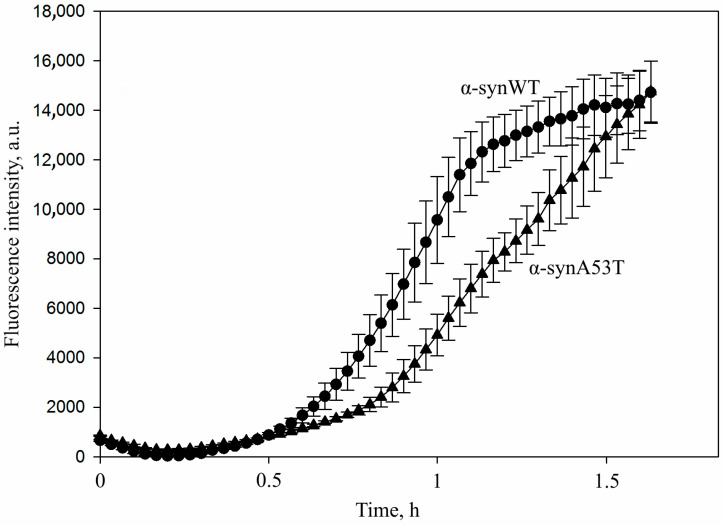
Amyloid transformation of α-synWT and its mutant form α-synA53T under conditions used for the generation of seeds. α-synWT (circles) or its mutant form α-synA53T (triangles) were incubated at a concentration of 1 mg/mL (70 µM) in the presence of 0.015% SDS in PBS buffer, pH 7.4, at 42 °C. The sample was mixed by orbital shaking at 400 rpm for 1 min, followed by a 1 min incubation without stirring. At least 35 such cycles were performed. The formation of beta-sheet structures was monitored by changes in thioflavin T fluorescence. Fibrillation kinetics were analyzed in a 96-well plate (Greiner, Kremsmuenster, Austria, non-binding, μClear^®^, black) with sealing film in 100 μL/well. The fluorescence was measured at 30 min intervals through the bottom of the plate using a CLARIOstar plate reader (BMG LABTECH GmbH, Germany). Data are presented as the mean ± SD of three independent measurements.

As could be seen from the data presented in Figure 1, the kinetics of the seeds’ formation differ for the α-synWT protein and its mutant form α-synA53T. In the case of the wild-type alpha-synuclein, the onset of the formation of oligomeric forms enriched in beta-strands occurred after 15 cycles (30 min), while mutant form A53T aggregation was characterized by a more prolonged lag phase. A similar pattern was observed at the end of incubation: for α-synWT the fluorescence intensity of thioflavin T reached a plateau after 35 cycles, whereas for α-synA53T the fluorescence intensity continued to increase exponentially. It should be noted that under standard conditions, the fibrillation of the alpha-synuclein A53T occurs more rapidly in contrast to the wild-type protein, probably due to conformational alterations in the mutant protein.

The properties of the obtained seeds of both proteins were studied using the dynamic light scattering (DLS) and transmission electron microscopy (TEM) methods. DLS data showed that 90% of amyloid aggregates obtained from wild-type alpha-synuclein had a hydrodynamic diameter of 20–40 nm, while in the case of A53T, 80% of the derivatives were 25–50 nm in size (Supplementary Figure S1). We also analyzed the half-life of the seeds to figure out how quickly they needed to be used. As a result, we applied them within half an hour after preparation to prevent the particles from agglutinating and forming larger aggregates. TEM did not reveal large fibrils or amorphous aggregates, but showed a relatively homogeneous set of small particles with a cross-sectional size of 11 ± 2 nm (Supplementary Figure S2).

**Figure 2:**
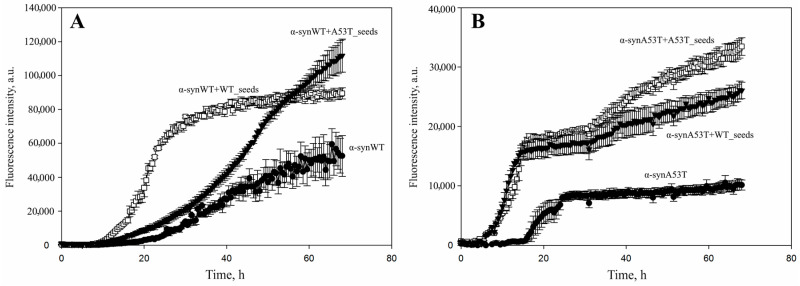
Amyloid transformation of (**A**) α-synWT and (**B**) its mutant form α-synA53T triggered by the addition of different types of amyloid seeds particles. A total of 50 μM of wild-type alpha-synuclein (**A**) or its mutant form A53T (**B**) was incubated without additives (black circles), in the presence of 1% wild-type seeds or A53T seeds in PBS buffer, pH 7.4 at 37 °C and constant stirring for 67 h. Amyloid aggregation of alpha-synuclein was detected by the change in thioflavin T fluorescence added in 10-fold excess. Fibrillation kinetics were analyzed in a 96-well plate (Greiner, Kremsmuenster, Austria, non-binding, μClear^®^, black) with sealing film in 100 μL/well. The fluorescence was measured at 30 min intervals through the bottom of the plate using a CLARIOstar plate reader (BMG LABTECH GmbH, Germany). Data are presented as the mean ± SD of three independent measurements.

Seeds obtained from two types of alpha-synuclein (wild-type and mutant form A53T) were used to stimulate the fibrillation of alpha-synuclein monomers. As shown in Figure 2A, the fibrillization of wild-type alpha-synuclein was significantly accelerated by the addition of seeds derived from the wild-type protein. The addition of α-synA53T seeds also accelerated the process, but to a lesser extent and with different kinetic parameters. Thus, that fibrillation curve differed slightly from the curve obtained for the mixture with wild-type protein seeds. After α-synA53T seeds were added, a flattened curve with a smaller slope (a slight increase in ThT fluorescence detected only after 18 h of incubation) was acquired. After the addition of α-synWT seeds, a boost in the level of ThT fluorescence intensity was observed after just 12 h of incubation, reaching a plateau after 24 h. Hence, the fibrillation of wild-type alpha-synuclein was effectively accelerated by the addition of wild-type protein seeds. At the same time, cross-seeding by the use of mutant form A53T seeds was not as productive in terms of the rate of amyloid particles formation as for the seeds from the wild-type protein.

For the alpha-synuclein A53T there were no significant differences in the fibrillation kinetics after the addition of α-synA53T or α-synWT seeds (Figure 2B). The kinetic curves had a similar shape and approximately the same slope. An increase in ThT fluorescence level was observed after just 5 h of sample incubation, and a plateau was reached much more quickly than in the case of wild-type alpha-synuclein. The increase in absolute fluorescence values upon the addition of seeds from both wild-type and A53T alpha-synuclein might indicate the more efficient formation of amyloid fibrils. It also could be due to the presence of the larger number of beta-strands available for binding to thioflavin T compared to the samples where protein incubated alone.

**Figure 3:**
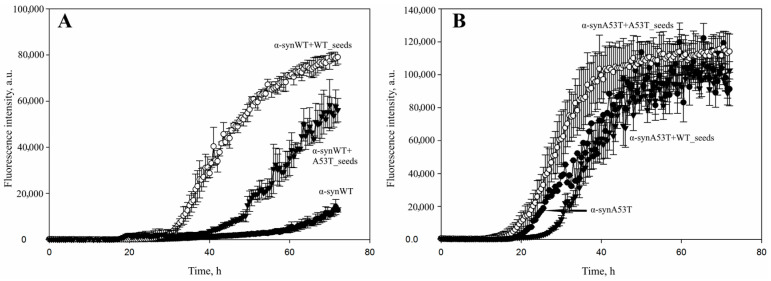
The effect of decreased concentrations of alpha-synuclein monomers on the stimulation of fibrillation triggered by the use of different types of alpha-synuclein seeds. (**A**) A total of 21 μM of wild-type alpha-synuclein was incubated without additives (black circles), in the presence of 1% wild-type seeds (white circles) or A53T seeds (black triangles). (**B**) A total of 21 μM of A53T mutant alpha-synuclein was incubated without additives (black circles), in the presence of 1% wild-type seeds (black triangles) or A53T seeds (white circles). Samples were incubated in PBS buffer, pH 7.4 at 37 °C and constant stirring for 72 h. Amyloid aggregation of alpha-synuclein protein was detected by the change in thioflavin T fluorescence added in 10-fold excess recording changes in ThT fluorescence every 30 min. Data are presented as the mean ± SD of three independent measurements.

Stimulation of fibrillation by the addition of different types of seeds was also observed after a 2.5-fold decrease in the concentration of alpha-synuclein monomers. Thus, when the experimental concentration of alpha-synuclein was reduced to 21 μM, the wild-type protein showed a fibrillation dynamic similar to the one observed in the case of a 50 μM concentration (Figure 2A and Figure 3A). However, for the alpha-synuclein A53T the results were slightly different: at first α-synA53T seeds were shown to trigger fibrillation process more effectively than the α-synWT seeds. Furthermore, the addition of wild-type alpha-synuclein seeds even slowed down fibrillation kinetics during the initial incubation period. Nevertheless, after 2 days of incubation the signal levels were approximately equal and remained within the statistical error (Figure 2B and Figure 3B).

**Figure 4:**
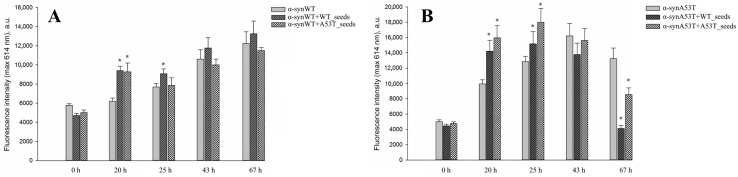
The changes in Congo red fluorescence intensity maximum in the presence of (**A**) α-synWT or (**B**) α-synA53T protein during their fibrillation without seeds and in the presence of different types of seeds. A total of 50 µM α-syn was incubated in PBS buffer, pH 7.4 for 67 h without seeds and in the presence of different types of seeds. Seeds were added to a final concentration of 1% relative to the target protein concentration in the sample. The samples were incubated at 37 °C with constant stirring. During fibrillation, aliquots were taken from tested samples and mixed with a tenfold excess of freshly prepared Congo red in the corresponding buffer. After 15 min of incubation at 20 °C, the fluorescence spectra of Congo red were recorded using a FluoroMax^®^-3 spectrofluorometer (Horiba Jobin Yvon, Palaiseau, France) over a wavelength range of 520–700 nm, with an excitation wavelength of 497 nm. Data are presented as the mean ± SD of three independent measurements. *—a statistically significant difference at the *p* ≤ 0.05 level (compared to the protein without additives).

Along with the detection of thioflavin T fluorescence, we confirmed the formation of amyloid fibrils by checking the changes in the fluorescence intensity of Congo red dye (Figure 4). Congo red fluorescence intensity is known to increase at 614 nm (excitation wavelength 497 nm) during the binding to amyloid particles.

We observed an increase in Congo red fluorescence intensity levels in all experimental samples during the incubation period, which corresponded to previously obtained data (ThT fluorescence) and also indicated the formation of amyloid structures. The sub-sequent decrease in fluorescence intensity at later time points was probably related to the aggregation of the formed particles which may prevent the incorporating of the dye be-tween the formed beta-strands due to steric hindrance. Although the detailed kinetics of the process in those experiments could be hard to detect, aggregation definitely occurred in all studied samples—it was confirmed by particle size analysis using the DLS method at the same time intervals (Table 1).

As shown in Table 1, the aggregation process occurred in all the samples studied and a gradual increase in the hydrodynamic diameter of particles (nm) was seen throughout the incubation. The effective formation of oligomeric forms was observed after 20 h. Moreover, in the case of the mutant form A53T the aggregation proceeded faster. Consequently, large particles formed during the process could no longer be detected by the DLS method due to their sedimentation, while in samples with wild-type alpha-synuclein monomeric forms could still be observed. Once again, seeds boosted protein aggregation, as was also well shown using Thioflavin T and Congo red fluorescence assays. Furthermore, the addition of α-synWT seeds led to the transformation of all wild-type protein into large particles (1000–3000 nm), whereas in samples with α-synA53T seeds wild-type alpha-synuclein was still present in monomeric form (particle diameters 1–5 nm; ~2000 nm)—this was also consistent with the previously obtained results. After 67 h of incubation, only small-sized particles, i.e., monomers and small oligomers, were detected in the solution, while large fibrils rapidly shifted to the bottom of the cuvette. Distribution curves of particles’ hydrodynamic diameter are shown in Figures S3–S8 in the Supplementary.

The structures of the resulting fibrils were studied using transmission electron microscopy (TEM). As we previously demonstrated, the transformation of wild-type alpha-synuclein monomers and its mutant form A53T under the fibrillation conditions resulted in the formation of severe fibrillar structures. In the case of the wild-type protein, fibrils approximately 200 nm in length were detected. They formed a reticulate structure without evident signs of folding or interaction between lateral surfaces. Meanwhile, in the case of the alpha-synuclein A53T, the formation of fibrils approximately 350 nm in length with clearly visible helical twists was revealed. A similar helical arrangement of protofibrils was also demonstrated in the previously published article [27]. The authors also determined the helical pitch, helical height, and the twist angle of the protofibrils relative to each other. In other words, a restructuring and rearrangement of the protein molecule of alpha-synuclein mutant form A53T occurs. This leads to the formation of fibrils that are extremely different in their structural characteristics compared to the fibrils of the wild-type protein.

The results of the electron microscopy data analysis are shown in Table 2.

**Figure 5:**
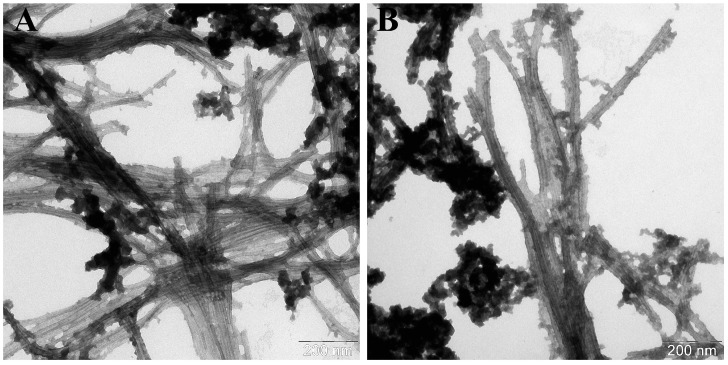
Electron micrographs of α-synWT aggregates formed in the presence of α-synWT seeds (**A**) in the field of view number 1 and (**B**) number 2. A total of 50 μM of α-synWT was incubated in the presence of 1% wild-type seeds in PBS, pH 7.4 at 37 °C and constant stirring for 67 h. The samples were stained with 1% uranyl acetate solution.

**Figure 6:**
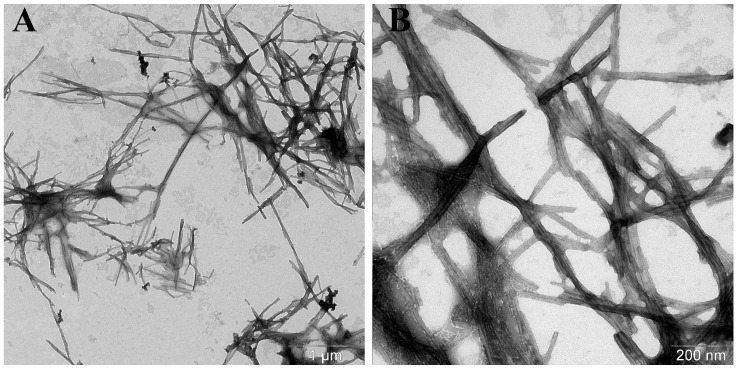
Electron micrographs of α-synWT aggregates formed in the presence of α-synA53T seeds (**A**) in the field of view number 1 and (**B**) number 2. A total of 50 μM of α-synWT was incubated in the presence of 1% A53T mutant form seeds in PBS, pH 7.4 at 37 °C and constant stirring for 67 h. The samples were stained with 1% uranyl acetate solution.

Based on the data presented in Table 2, it appeared that the incubation of wild-type alpha-synuclein with its seeds resulted in the formation of fibrils of greater length (600 nm vs. ~200 nm) and width (12.9 ± 1.1 vs. 10 ± 1 nm) compared to the α-synWT fibrils. In addition, transmission electron microscopy images (Figure 5) showed that these fibrils were tightly packed into stacks and lay parallel to each other along the whole length contacting their lateral surfaces. That pattern might be observed due to their hydrophobic properties. On the other hand, adding α-synA53T seeds to the samples containing wild-type alpha-synuclein led to the increase in the length of the fibrils (˃700 nm) and to the decrease in their width (10 ± 1 nm vs. 5.4 ± 1.0 nm). Also, the first signs of spiral twisting of the protofibrils in a fibril composition were noted (Figure 6B).

**Figure 7:**
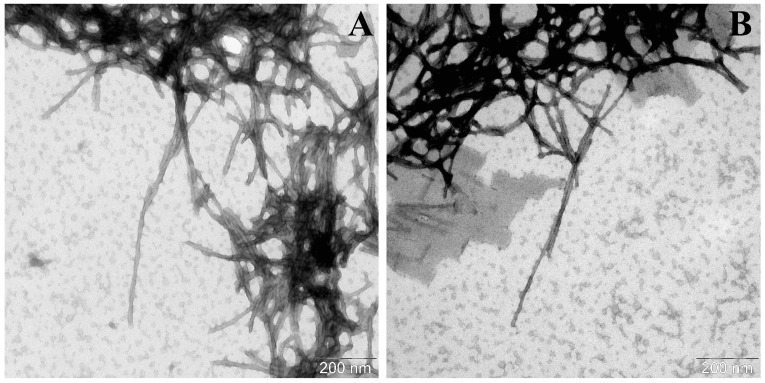
Electron micrographs of α-synA53T aggregates formed in the presence of α-synA53T seeds (**A**) in the field of view number 1 and (**B**) number 2. A total of 50 μM of α-synA53T was incubated in the presence of 1% A53T mutant form seeds in PBS, pH 7.4 at 37 °C and constant stirring for 67 h. The samples were stained with 1% uranyl acetate solution.

**Figure 8:**
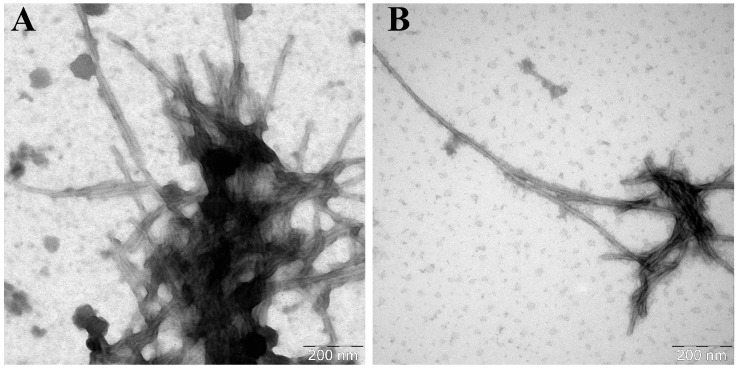
Electron micrographs of α-synA53T aggregates formed in the presence of α-synWT seeds (**A**) in the field of view number 1 and (**B**) number 2. A total of 50 μM of α-synA53T was incubated in the presence of 1% wild-type seeds in PBS, pH 7.4 at 37 °C and constant stirring for 67 h. The samples were stained with 1% uranyl acetate solution.

In the case of alpha-synuclein A53T, fibrils with a greater average length and smaller diameter were formed compared to the wild-type protein (Table 2). These fibrils were often spirally twisted relative to each other: their protofibrils folded into braided patterns. Similar to the experiments with the wild-type alpha-synuclein, addition of the seeds induced the formation of longer fibrils. Moreover, any variant of seeds, regardless of their source, caused the onset of wider fibrils, compared to the α-synA53T fibrils matured without additives (Figure 7 and Figure 8).

## 4. Discussion

In recent years, the use of RT-QuIC (real-time quaking-induced conversion) as a diagnostic tool for detecting neurodegenerative diseases has expanded. Initially, RT-QuIC was used to diagnose prion diseases, as they are characterized by the induction of the transformation of normal prion protein monomers upon their interaction with the infectious form [30,31]. This method began to be used for diagnosing synucleinopathies when it became clear that alpha-synuclein had the ability to undergo prion-like amyloid transmission when the aberrant protein interacts with its normal form [18,32]. A similar system has been applied, but less widely, for the diagnosis of tauopathies of various etiologies [33,34,35].

RT-QuIC is most widely used for the diagnosis of synucleinopathies, as these diseases—and Parkinson’s disease in particular—are of significant public health concern. However, detecting aberrant forms of alpha-synuclein in cerebrospinal fluid requires very time-consuming and therefore expensive experiments. This outcome should be due to the slow progression of amyloid transformation of the alpha-synuclein monomers used as a substrate when they interact with aberrant forms of the protein, which are presented in biological fluid samples. In addition, ambiguous assay results could be related to the peculiarities of cross-seeding between different forms of alpha-synuclein. For example, the amyloid transformation of substrate alpha-synuclein monomers may occur differently in the presence of aberrant forms of wild-type alpha-synuclein or mutant forms that characterize sporadic and hereditary forms of Parkinson’s disease, respectively.

The cross-seeding between alpha-synuclein and its mutant forms has been used in a number of studies to address various problems, but the obtained results seem to be quite scattered [36,37,38]. For example, it has been shown that cross-seeding of mutant and wild-type alpha-synuclein appeared to reduce aggregation efficiency in vitro, yet some mutant forms of alpha-synuclein may, on the contrary, increase the accumulation of amyloid structures in the brains of transgenic animals in in vivo experiments [39,40]. Yet, mostly wild-type alpha-synuclein monomers are used in RT-QuIC assays regardless of the type of synucleinopathy being tested for.

In our study, we showed that seeds derived from both types of alpha-synuclein (wild-type and A53T) significantly improve the effectiveness of the amyloid transformation of their relevant monomers and that our modified method to obtain seeds allowed us to model the amyloid transformation of substrate alpha-synuclein monomers. During the preparation of such alpha-synuclein seeds, designed for use as a priming agent, it is important to choose conditions under which small amyloid particles are formed. It is essential for maximizing the number of fibrillation centers with a minimum amount of protein. These conditions, namely, the use of detergent and elevated temperature, were optimized for obtaining seeds from wild-type alpha-synuclein and its mutant form A53T. The preparation of proper seeds was confirmed using the dynamic light scattering and transmission electron microscopy methods.

But still, this process depended on the specific pairs of proteins used in the experiments. Efficient fibrillation occurred when α-synWT seeds were added to wild-type alpha-synuclein monomers. In contrast, during the incubation of α-synA53T seeds with wild-type alpha-synuclein monomers the lag phase significantly increased, and the fibrillation efficiency returned to the one observed in the sample without seeds. This allowed us to assume that detecting aberrant mutant forms of alpha-synucleins in biological fluids might be challenging, if wild-type alpha-synuclein monomers are used in test systems.

Our experimental data showed that crucial advantages throughout testing could be achieved by applying monomers of alpha-synuclein mutant forms, particularly the α-synA53T variant. This mutant form of alpha-synuclein is characterized by the rapid formation of amyloid structures, manifested in a pronounced decrease in lag phase duration. Mixing either of the two types of seeds with alpha-synuclein A53T monomers further enhances the rate of fibril formation, making the use of this particular mutant optimal for designing diagnostic test systems. Moreover, under certain conditions, unique effects of various seed types on the α-synA53T fibrillation kinetics could be revealed.

Thus, in our study, we compared under identical conditions the efficacy of the seeding and cross-seeding of wild-type and A53T mutant alpha-synuclein monomers in the amyloid transformation reaction with two types of seeds. Here the seeds served as the mode for two types of aberrant alpha-synuclein forms that may be present in biological fluid samples from patients with synucleinopathies. The testing system itself was similar to the one used in RT-QuIC. We were able to clearly demonstrate that the use of the alpha-synuclein A53T mutant form increased the efficiency of the process and, more importantly, accelerated the analysis while using any type of alpha-synuclein aggregate.

We think that using different mutant forms of alpha-synuclein as monomers will speed up the testing process. In addition, our approach could allow the development of differentiated test systems to not only detect aberrant forms of alpha-synuclein in biological fluids but also to determine the specific mutant forms of protein responsible for this process. Obviously, developing such test systems will require obtaining different mutant forms of alpha-synuclein associated with specific synucleinopathies. This further implies careful testing of the hypotheses made with both individual proteins and cerebrospinal fluid samples that came from specific patients.

We believe that this information may be useful for researchers who are already working with RT-QuIC. Even at this stage, they could use mutant forms of alpha-synuclein to significantly accelerate analysis and to assess the possible causes of ambiguous results, which are often encountered when using RT-QuIC.

## 5. Conclusions

• Fibrillation of the alpha-synuclein A53T is accelerated by the addition of both wild-type and mutant alpha-synuclein seeds, which may facilitate the detection of various aberrant protein conformations in biological fluids.
• The low efficiency of wild-type alpha-synuclein fibrillation in the presence of alpha-synuclein A53T seeds may suggest that the features of cross-seeding cause a decrease in the effectiveness of current assays.
• The monomers of alpha-synuclein mutant form A53T are a promising candidate for use in test systems due to the significantly reduced lag period of fibrillation.

## Figures and Tables

**Table 1:** Hydrodynamic diameter of particles (nm) in the samples obtained during the fibrillation of different forms of alpha-synuclein both in the absence of seeds and in the presence of different types of seeds.

	0 h	2 h	4 h	6 h	20 h	25 h	46 h	67 h
α-synWT	2–7	4–5	4–5	1–5	2–6	1000–2500	1000–2000	3–16
					3000			
α-synWT + WT_seeds	5–8	5–6	5–6	2–8	1–2	1–2	1–2	1–2
		2000	18–20	20	1000–3000	300–800	15–16	4–8
			2000–3000				100–200	
							600–700	
α-synWT + A53T_seeds	1–7	6–8	4–6	2–8	1–5	200–1300	1–6	1–2
				13–18	~2000		~300	
α-synA53T	1–3	5–6	3–4	1–8	1–3	800–2500	1500–4200	600–1500
α-synA53T + A53T_seeds	1–7	2000–3000	~3000	>5000	1–7	1–2	700–3000	~10
						300–800		800–2000
α-synA53T + WT_seeds	~2;	10–12	~100	2000–5000	3000–4000	500–1800	200–1300	2000–4000
	2000–3000		~1000					

**Table 2:** Structural characteristics of wild-type and A53T alpha-synuclein fibrils prepared in the presence of seeds following 67 h of incubation obtained from TEM images (Figure 5, Figure 6, Figure 7 and Figure 8).

	Average Length, nm	Average Width ± SD, nm
α-synWT [27]	~200	10.0 ± 1
α-synWT + WT_seeds	600 ± 50	12.9 ± 1.1
α-synWT + A53T_seeds	770 ± 70	5.4 ± 1.0
α-synA53T [28,29]	~350	6.0 ± 0.6
α-synA53T + A53T_seeds	550 ± 40	9.6 ± 1.1
α-synA53T + WT_seeds	1800 ± 140	11.7 ± 1.1

## Data Availability

The original contributions presented in this study are included in the article/Supplementary Material. Further inquiries can be directed to the corresponding author.

## Supplementary Materials

The following supporting information can be downloaded at: https://www.mdpi.com/article/10.3390/life16040675/s1. Figure S1: Hydrodynamic diameter of particles in samples obtained during the generation of alpha-synuclein seeds; Figure S2: Electron micrographs of α-synWT (a) and α-synA53T (b) seeds; Figure S3: Hydrodynamic diameter of particles in samples obtained during the wild-type alpha-synuclein (α-synWT) amyloid aggregation; Figure S4: Hydrodynamic diameter of particles in samples obtained during the mutant A53T alpha-synuclein (α-synA53T) amyloid aggregation; Figure S5: Hydrodynamic diameter of particles in samples obtained during the wild-type alpha-synuclein (α-synWT) amyloid aggregation in the presence of α-synWT seeds; Figure S6: Hydrodynamic diameter of particles in samples obtained during the wild-type alpha-synuclein (α-synWT) amyloid aggregation in the presence of α-synA53T seeds; Figure S7: Hydrodynamic diameter of particles in samples obtained during the mutant A53T alpha-synuclein (α-synA53T) amyloid aggregation in the presence of α-synA53T seeds; Figure S8: Hydrodynamic diameter of particles in samples obtained during the mutant A53T alpha-synuclein (α-synA53T) amyloid aggregation in the presence of α-synWT seeds.

## Abbreviations

The following abbreviations are used in this manuscript:

α-synWT	recombinant human wild-type alpha-synuclein
α-synA53T	mutant form of recombinant human alpha-synuclein with replacement of the 53d alanine residue with a threonine one
CSF	cerebrospinal fluid
DLS	dynamic light scattering
