# Supplementary material for: Features of Cross-Seeding of Wild-Type Alpha-Synuclein and Its Mutant Form A53T Potentially Useful for the Development of Test Systems
Source: Life (Basel). 2026 Apr 15;16(4):675. doi: 10.3390/life16040675 (PMC13117027; doi:10.3390/life16040675)
Supplement: Supplementary file 1 [file life-16-00675-s001.zip › life-4226038-supplementary.pdf]

# *Supplementary Material*

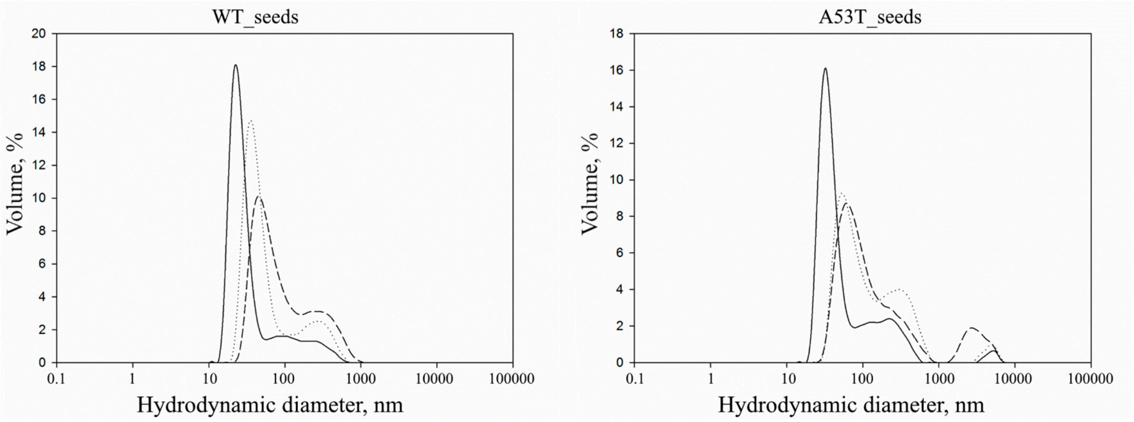


**Figure S1.** Hydrodynamic diameter of particles in samples obtained during the generation of alpha-synuclein seeds.

*α-synWT (left panel) or its mutant form α-synA53T (right panel) were incubated at a concentration of 1 mg/mL (70 µM) in the presence of 0.015% SDS in PBS buffer, pH 7.4, at 42°C. The sample was mixed by orbital shaking at 400 rpm for 1 min, followed by a 1-min incubation without stirring. At least 35 such cycles were performed. At the end of incubation, samples were collected and then studied using dynamic light scattering method.*

*The solid line indicates the particle distribution immediately after the seeds were prepared. The dotted line indicates the distribution 30 minutes later, and the dashed line - 1 hour later. We studded the half-life of the seeds to figure out how quickly they needed to be used. As a result, we applied them within half an hour after preparation to prevent the particles from agglutinating and forming larger aggregates.*

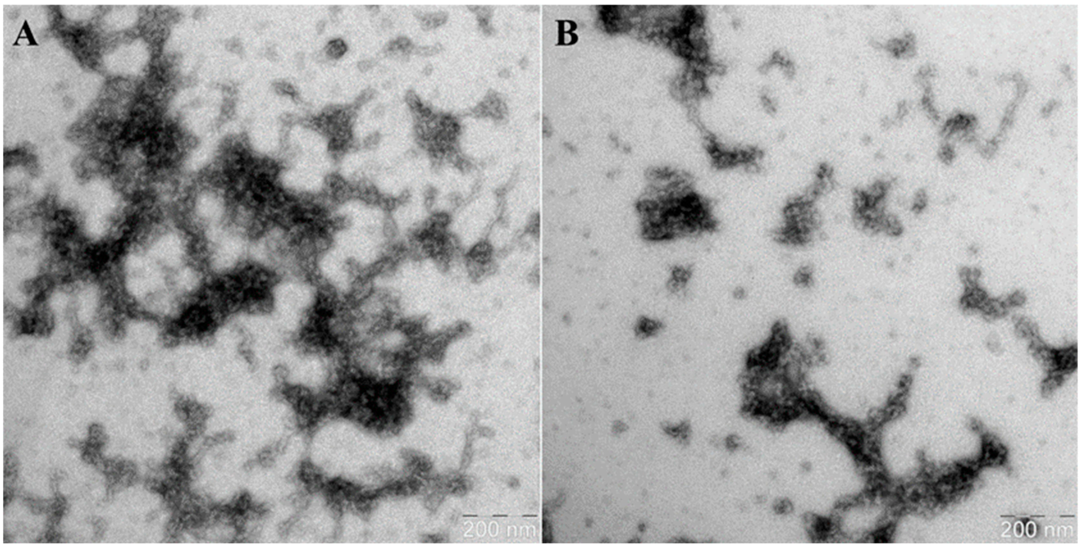


**(a)** **(b)**

**Figure S2.** Electron micrographs of α-synWT (a) and α-synA53T (b) seeds.

*α-synWT (left panel) or its mutant form α-synA53T (right panel) were incubated at a concentration of 1 mg/mL (70 µM) in the presence of 0.015% SDS in PBS buffer, pH 7.4, at 42°C. The sample was mixed by orbital shaking at 400 rpm for 1 min, followed by a 1-min incubation*

*without stirring. At least 35 such cycles were performed. At the end of incubation, samples were stained with 1% uranyl acetate solution.*

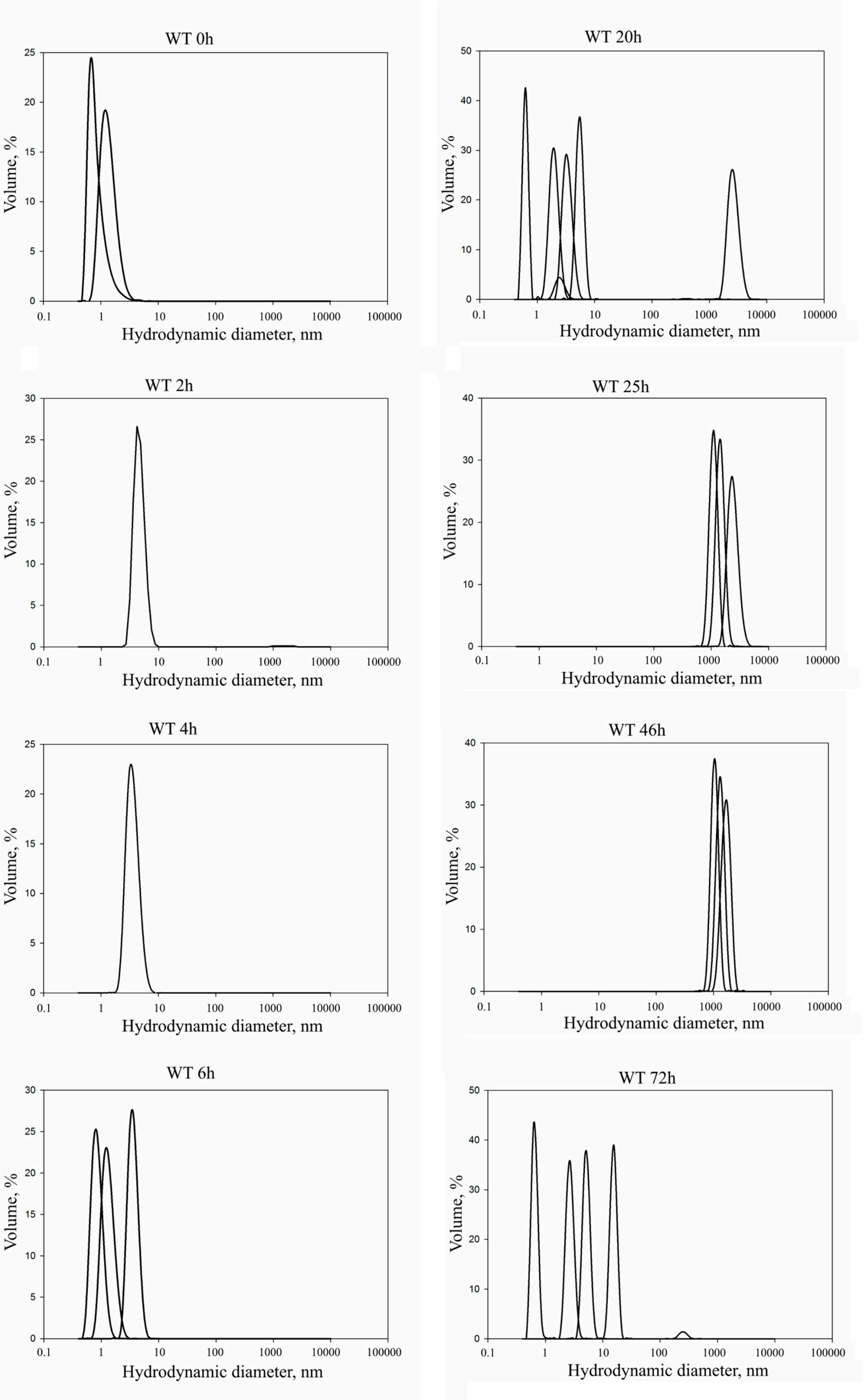


**Figure S3.** Hydrodynamic diameter of particles in samples obtained during the wild-type alpha-synuclein ($\alpha$-synWT) amyloid aggregation.

*50 μM of α-synWT was incubated in PBS buffer, pH 7.4 at 37ºC with constant orbital shaking at 600 rpm for 72 hours. Samples were collected at certain time points and then studied using dynamic light scattering method.*

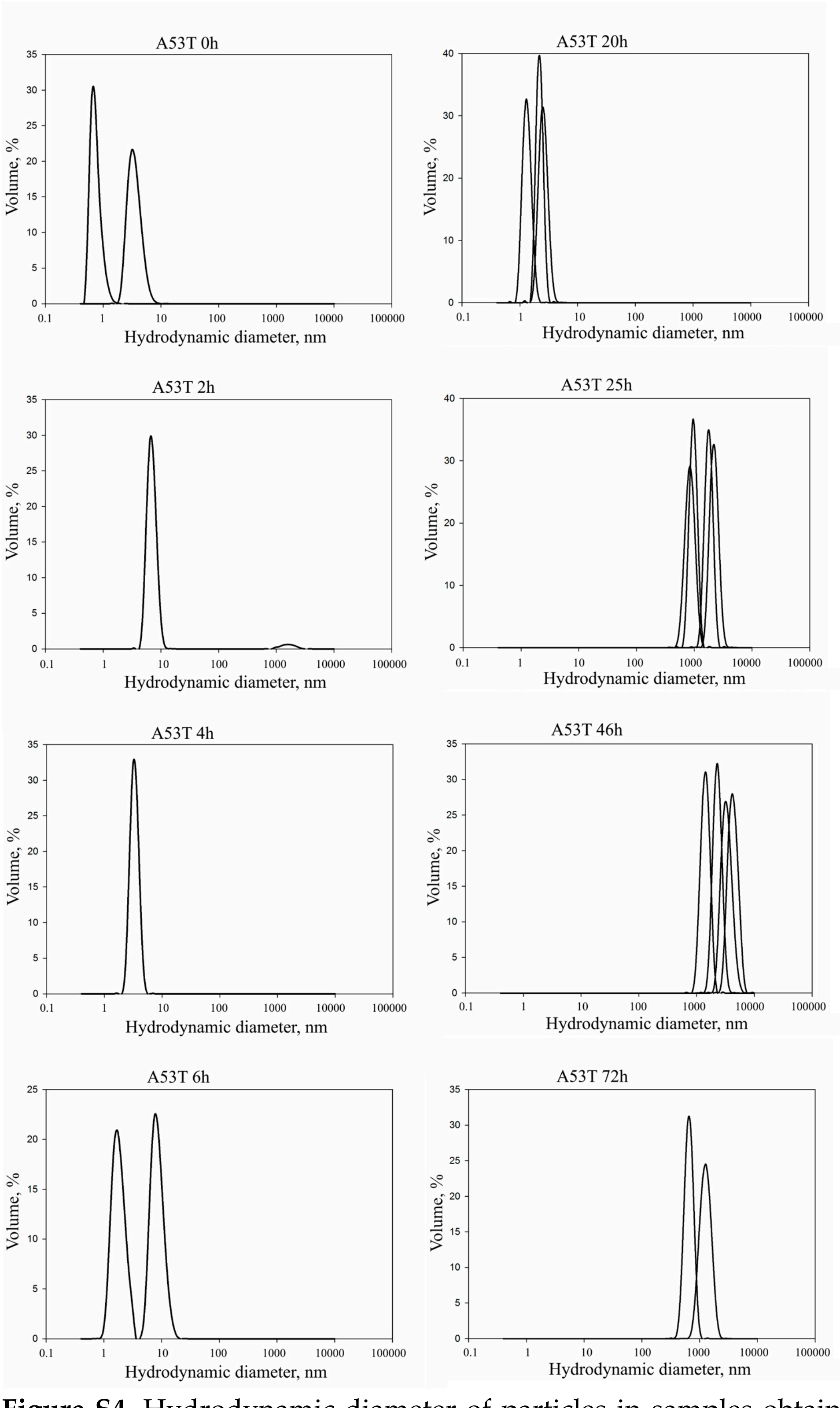


**Figure S4.** Hydrodynamic diameter of particles in samples obtained during the mutant A53T alpha-synuclein (α-synA53T) amyloid aggregation.

*50 µM of α-synA53T was incubated in PBS buffer, pH 7.4 at 37ºC with constant orbital shaking at 600 rpm for 72 hours. Samples were collected at certain time points and then studied using dynamic light scattering method.*

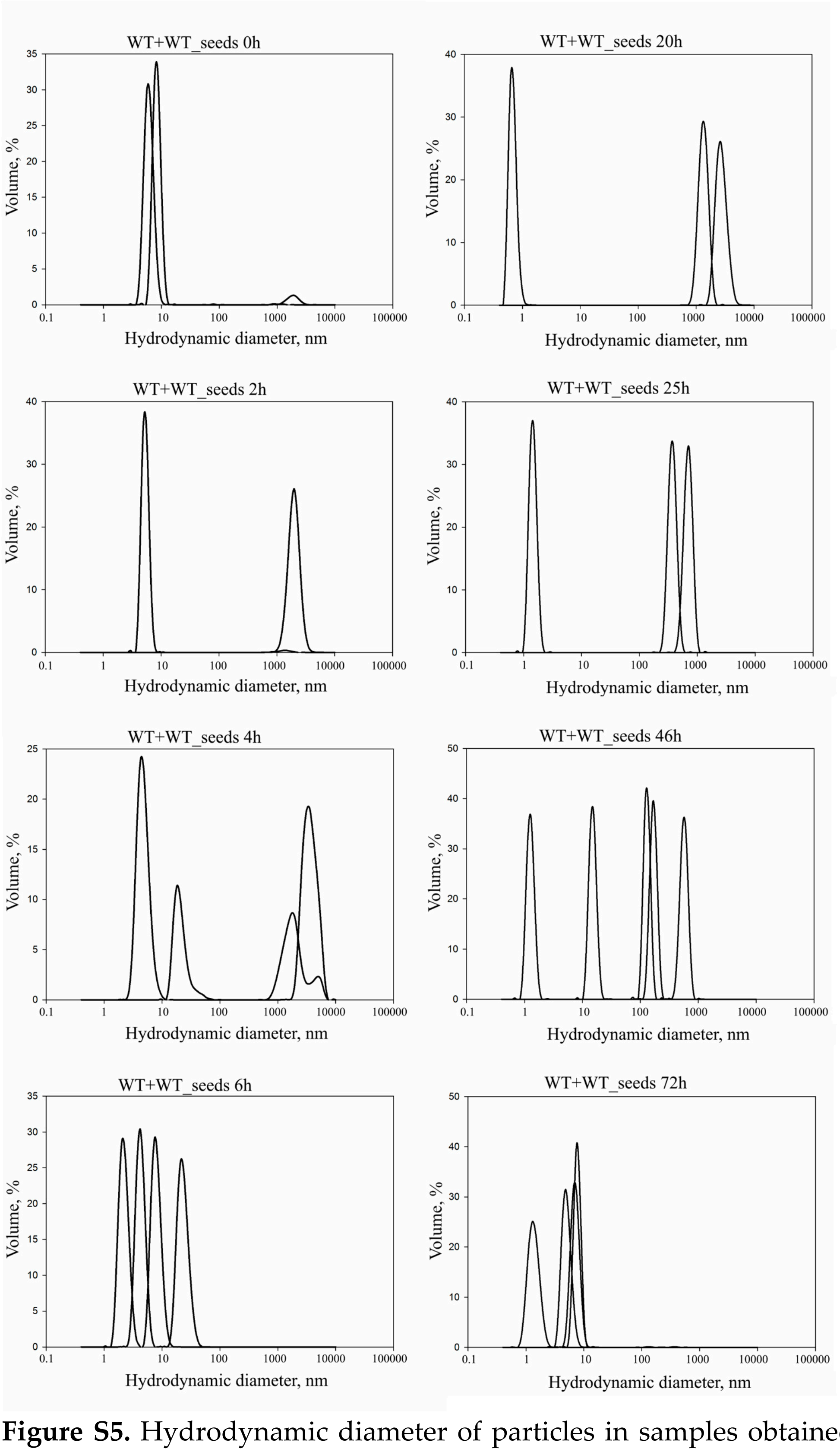


**Figure S5.** Hydrodynamic diameter of particles in samples obtained during the wild-type alpha-synuclein (α-synWT) amyloid aggregation in the presence of α-synWT seeds.

*50 µM of α-synWT was incubated in the presence of 0,5 µM α-synWT seeds (1%) in PBS buffer, pH 7.4 at 37ºC with constant orbital shaking at 600 rpm for 72 hours. Samples were collected at certain time points and then studied using dynamic light scattering method.*

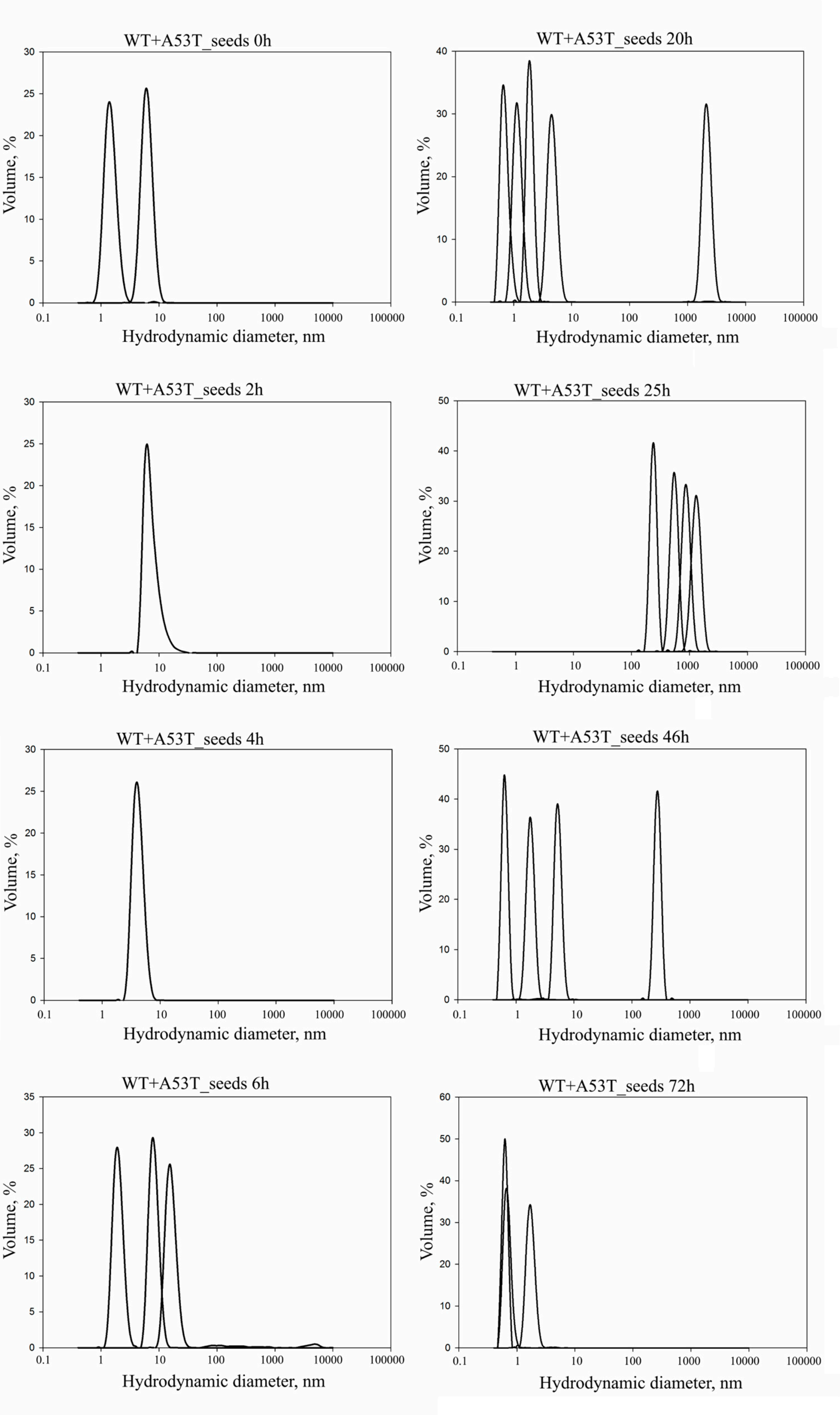


**Figure S6.** Hydrodynamic diameter of particles in samples obtained during the wild-type alpha-synuclein (α-synWT) amyloid aggregation in the presence of α-synA53T seeds.

*50 μM of α-synWT was incubated in the presence of 0,5 μM α-synA53T seeds (1%) in PBS buffer, pH 7.4 at 37ºC with constant orbital shaking at 600 rpm for 72 hours. Samples were collected at certain time points and then studied using dynamic light scattering method.*

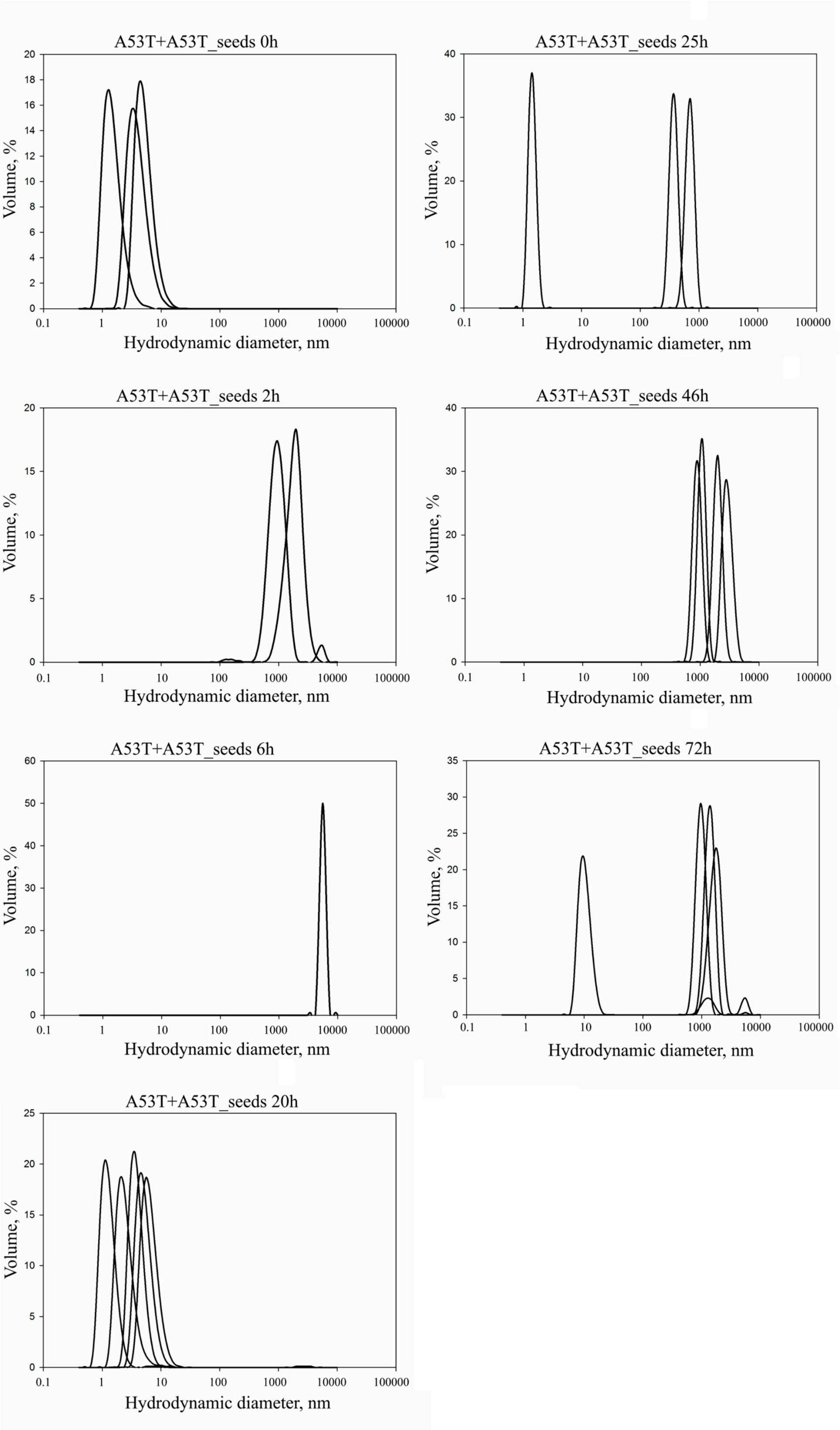


**Figure S7.** Hydrodynamic diameter of particles in samples obtained during the mutant A53T alpha-synuclein (α-synA53T) amyloid aggregation in the presence of α-synA53T seeds.

*50 µM of α-synA53T was incubated in the presence of 0,5 µM α-synA53T seeds (1%) in PBS buffer, pH 7.4 at 37ºC with constant orbital shaking at 600 rpm for 72 hours. Samples were collected at certain time points and then studied using dynamic light scattering method.*

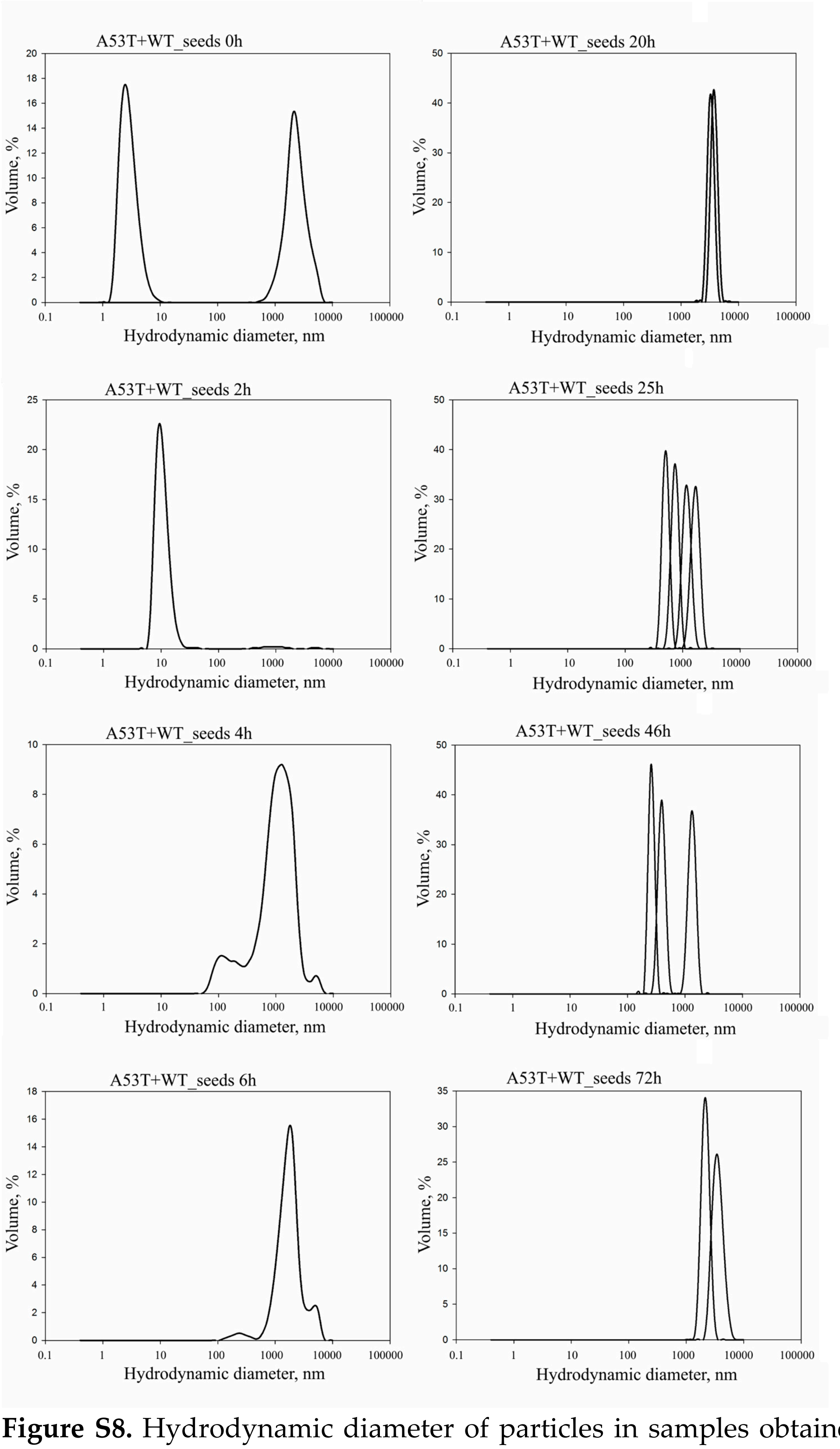


**Figure S8.** Hydrodynamic diameter of particles in samples obtained during the mutant A53T alpha-synuclein (α-synA53T) amyloid aggregation in the presence of α-synWT seeds.

*50 μM of α-synA53T was incubated in the presence of 0,5 μM α-synWT seeds (1%) in PBS buffer, pH 7.4 at 37ºC with constant orbital shaking at 600 rpm for 72 hours. Samples were collected at certain time points and then studied using dynamic light scattering method.*
