# Supplementary figures and images for: Features of Cross-Seeding of Wild-Type Alpha-Synuclein and Its Mutant Form A53T Potentially Useful for the Development of Test Systems
Source: Life (Basel). 2026 Apr 15;16(4):675. doi: 10.3390/life16040675 (PMC13117027; doi:10.3390/life16040675)

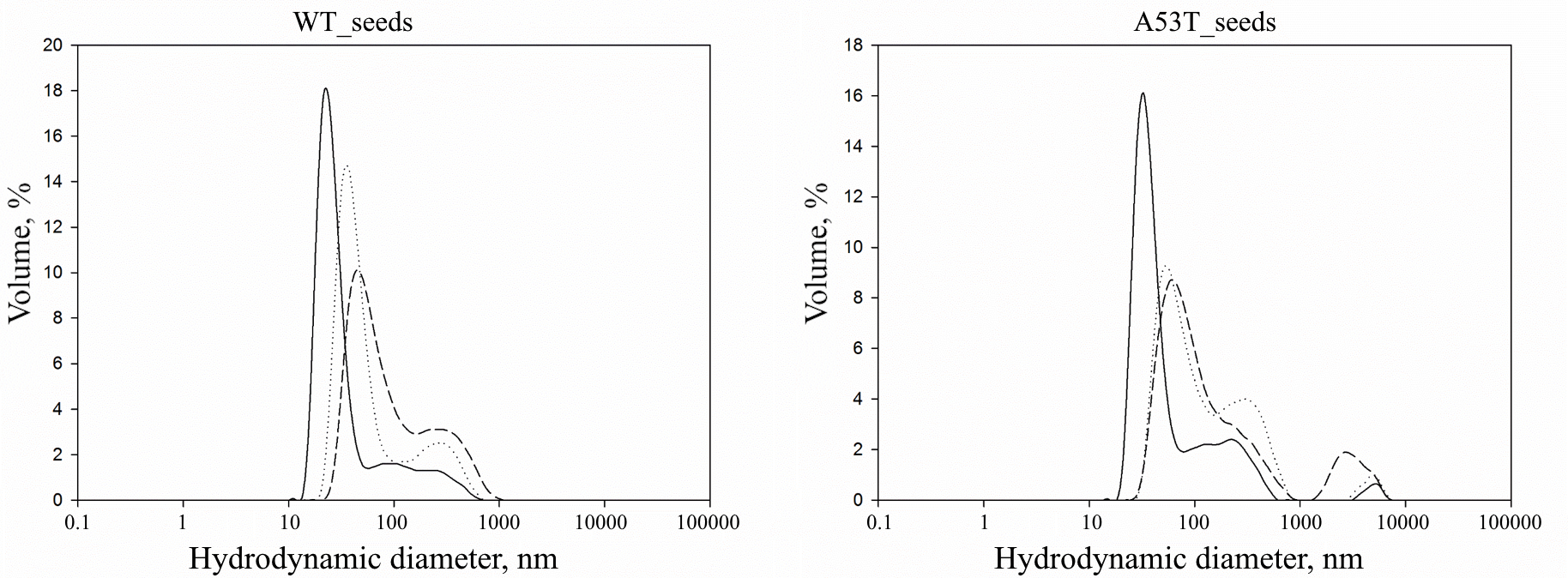

Supplement: Supplementary file 1 [file life-16-00675-s001.zip › Fig_S1_suppl_new.tiff]

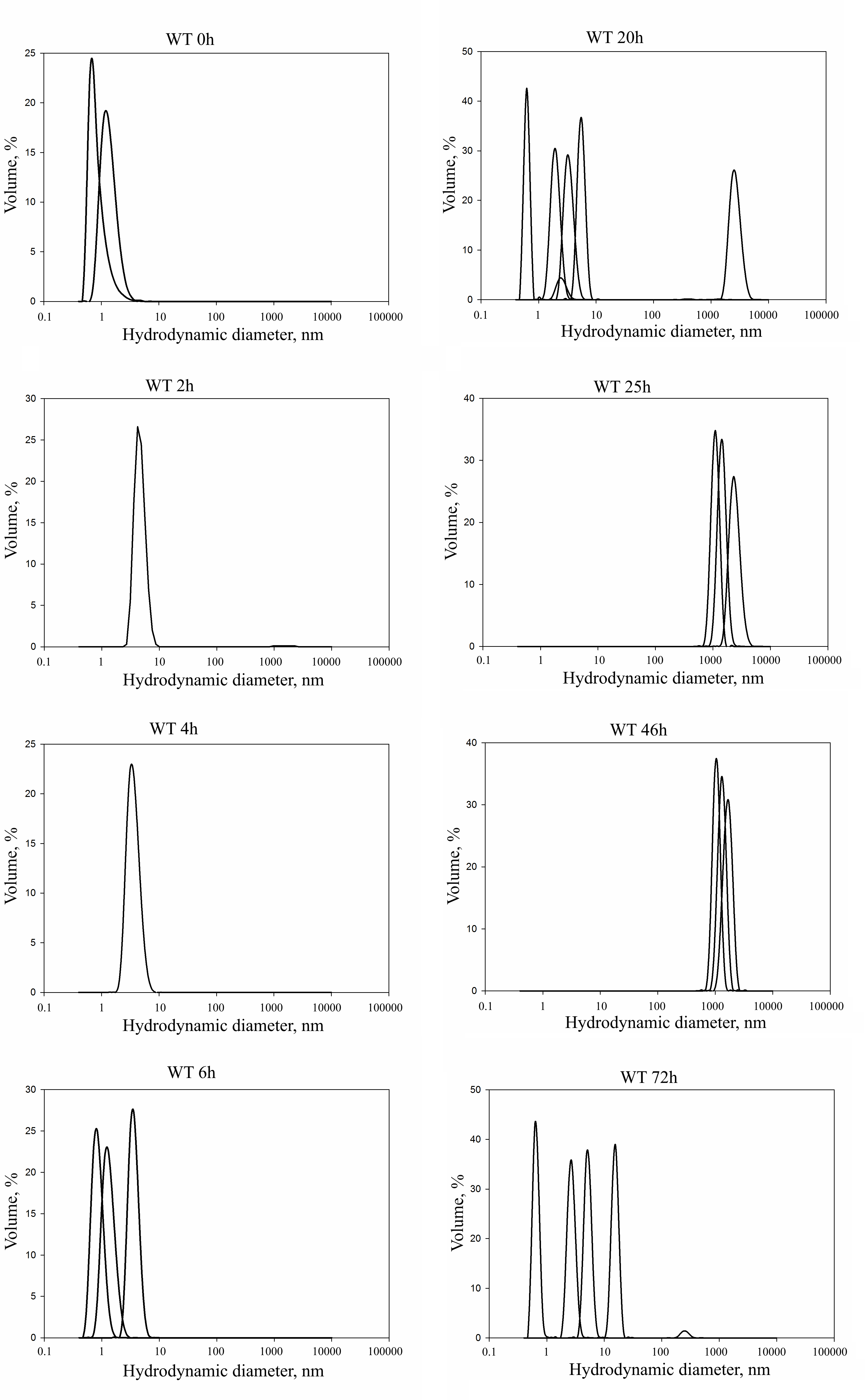

Supplement: Supplementary file 1 [file life-16-00675-s001.zip › Fig_S3_suppl_new.tiff]

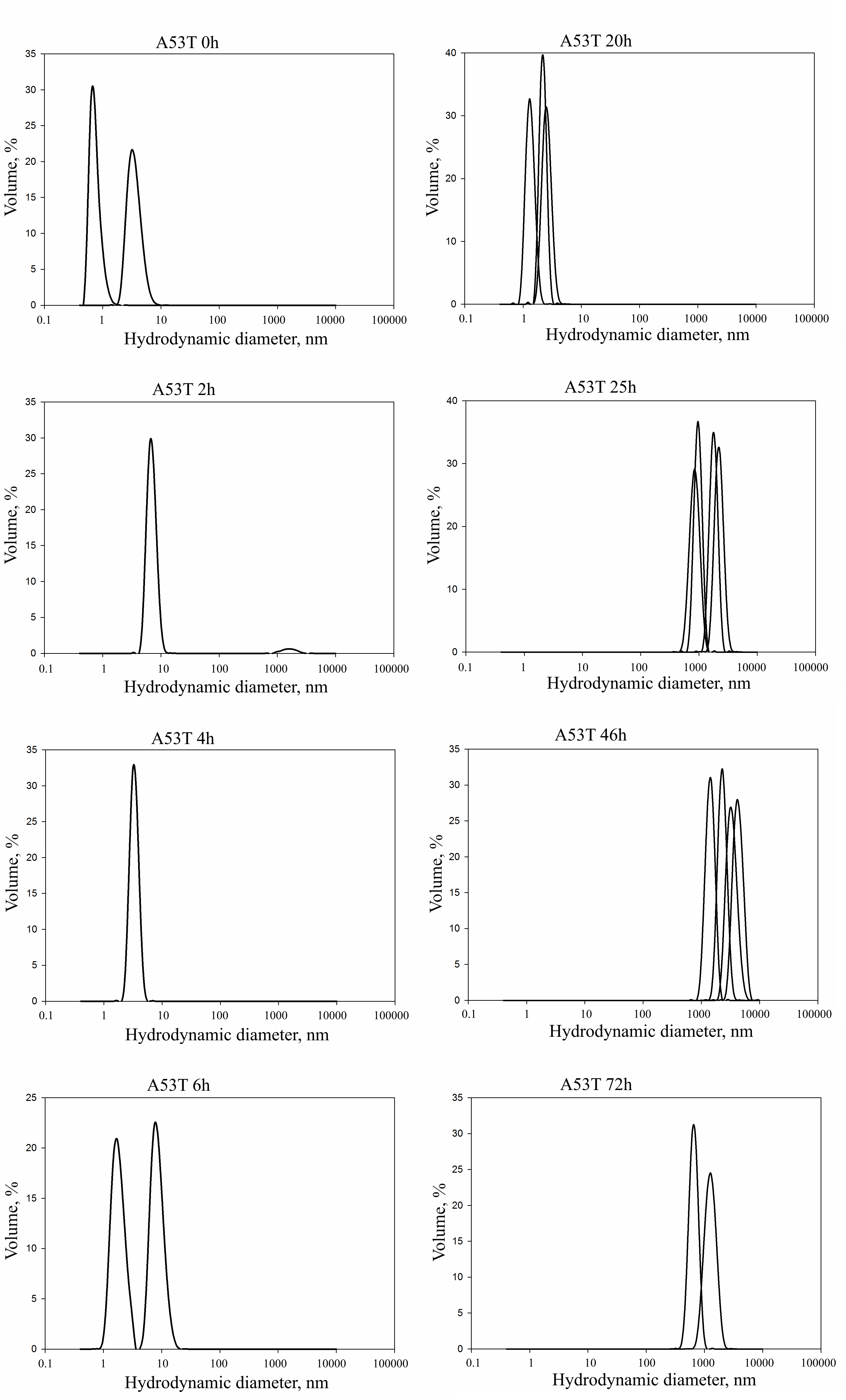

Supplement: Supplementary file 1 [file life-16-00675-s001.zip › Fig_S4_suppl_new.tiff]

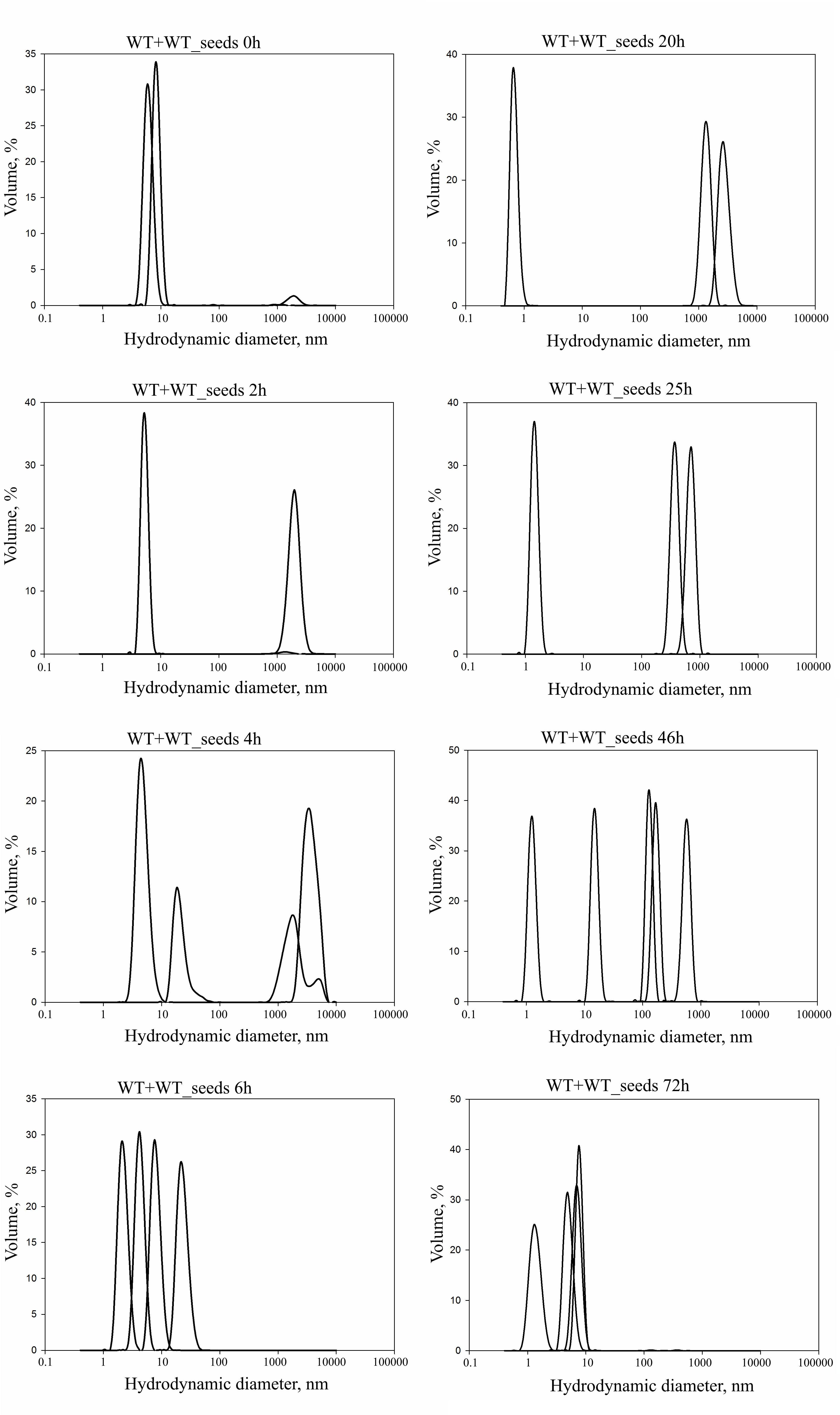

Supplement: Supplementary file 1 [file life-16-00675-s001.zip › Fig_S5_suppl_new.tiff]

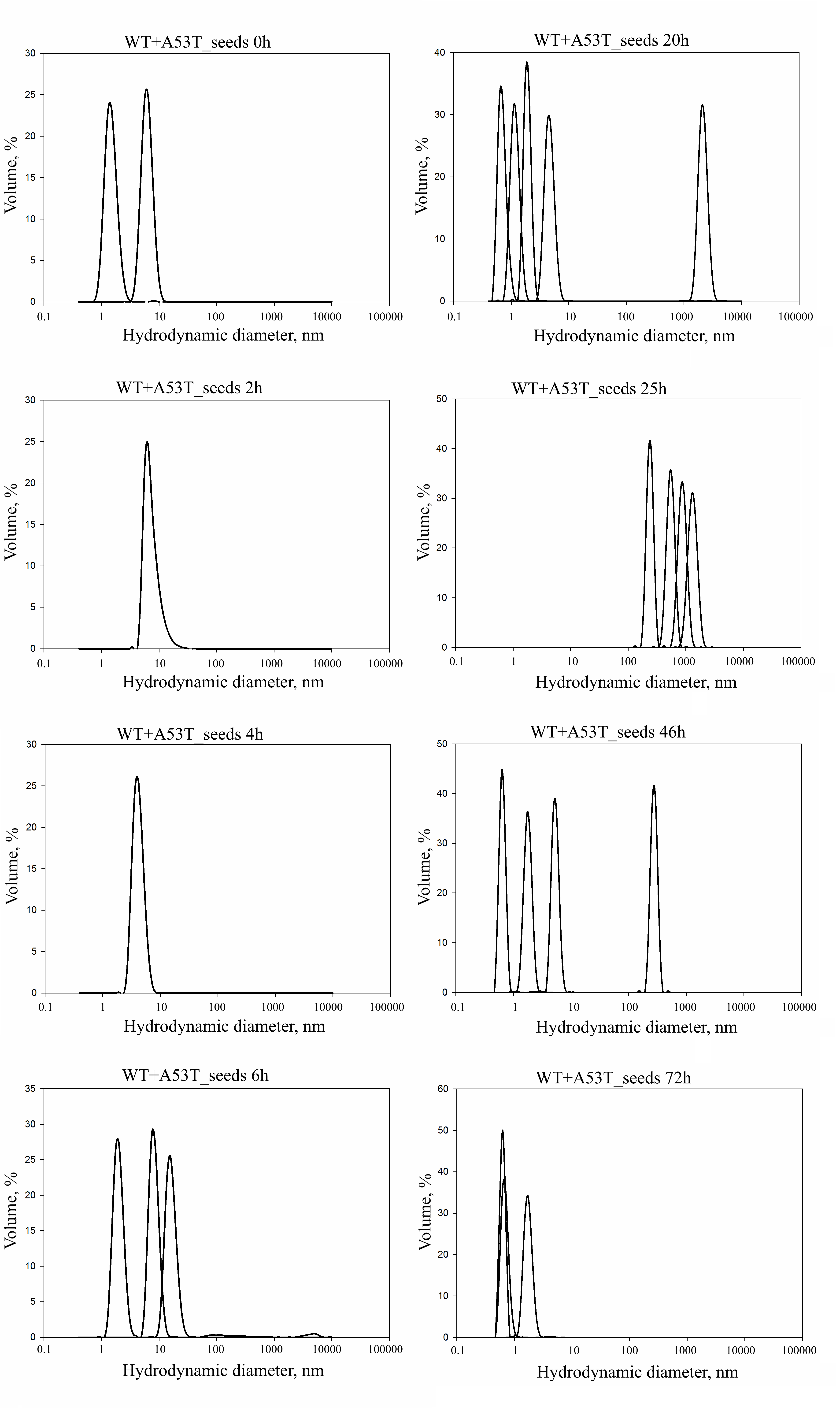

Supplement: Supplementary file 1 [file life-16-00675-s001.zip › Fig_S6_suppl_new.tiff]

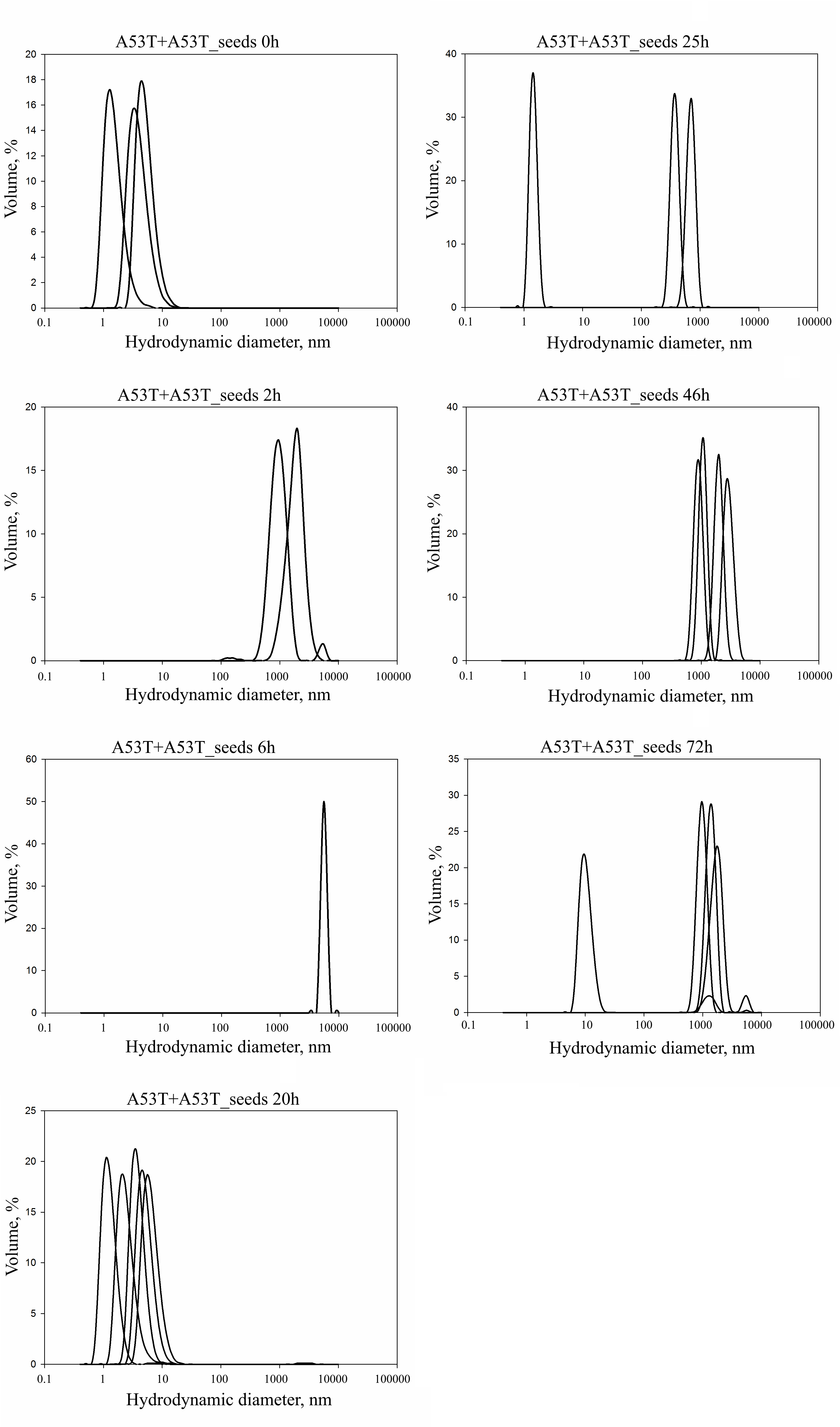

Supplement: Supplementary file 1 [file life-16-00675-s001.zip › Fig_S7_suppl_new.tiff]

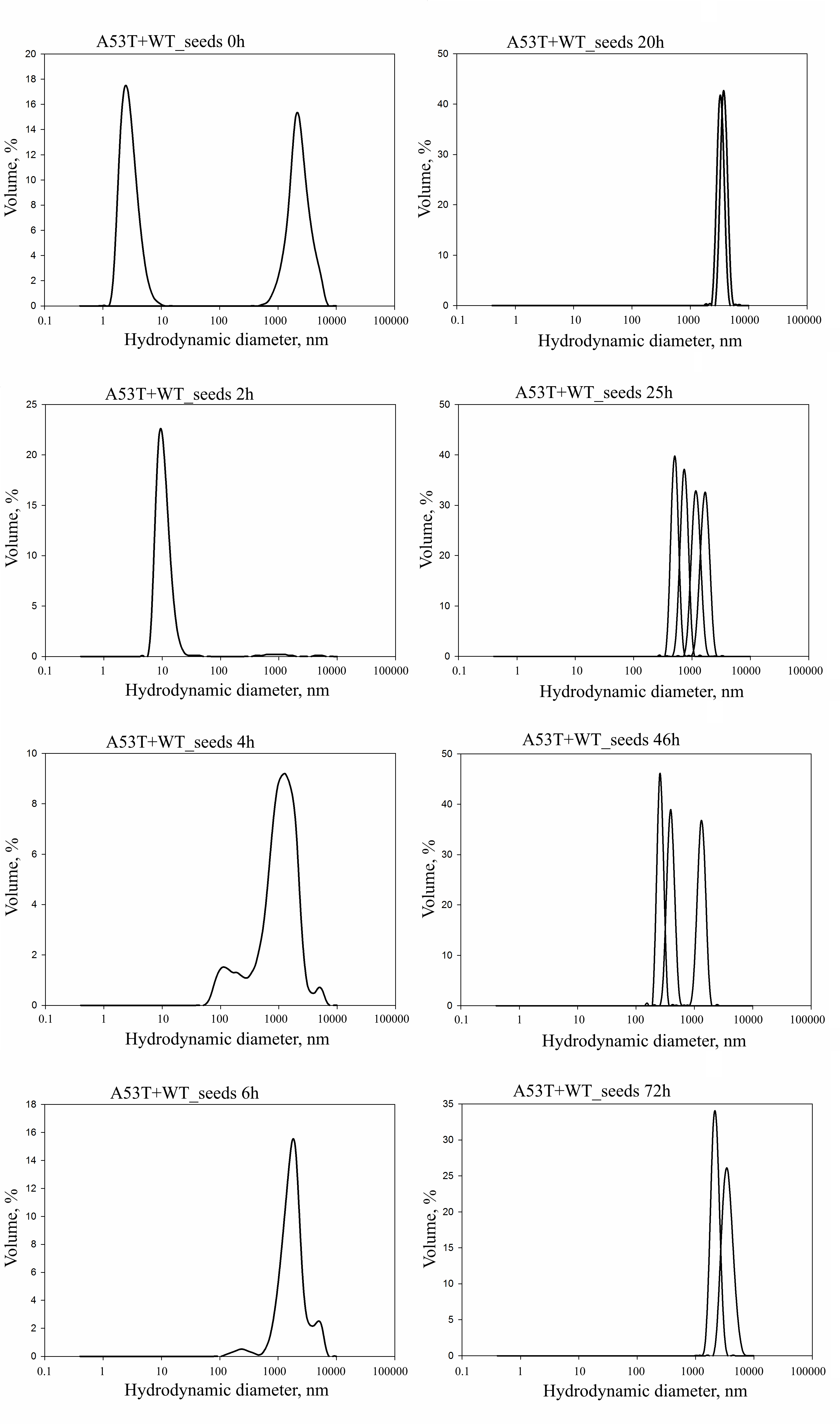

Supplement: Supplementary file 1 [file life-16-00675-s001.zip › Fig_S8_suppl_new.tiff]
